# From Medical Herb to Functional Food: Development of a Fermented Milk Containing Silybin and Protein from Milk Thistle

**DOI:** 10.3390/foods12061308

**Published:** 2023-03-19

**Authors:** Yanxia Liu, Minghuo Wu, Miaomiao Ren, Haijun Bao, Qing’an Wang, Nan Wang, Shibo Sun, Jianqiang Xu, Xiaojing Yang, Xu Zhao, Yongming Bao, Gaohong He, Weiping Xu

**Affiliations:** 1 School of Ocean Science and Technology, Panjin Institute of Industrial Technology, Dalian University of Technology, Panjin Campus, Panjin 124221, China; 2 Yingkou Chenguang Extraction Equipment Co., Ltd., Yingkou 115000, China; 3 School of Life and Pharmaceutical Sciences, Dalian University of Technology, Panjin Campus, Panjin 124221, China; 4 State Key Laboratory of Fine Chemicals, School of Chemical Engineering, Dalian University of Technology, Dalian 116024, China

**Keywords:** milk thistle, silybin, protein, NaHCO_3_ treatment, microbial fermentation, medical herb, functional foods

## Abstract

Milk thistle is a traditional medicinal herb. Silybin is a medicinal component found in the seed coat of milk thistle, which has liver-protective and anti-cancer properties. Conventional studies have focused on the extraction of silybin with organic reagents, which was inapplicable to the food industry. This study aims to develop a fermented milk containing silybin and protein from the milk thistle seeds. A three step procedure was developed, comprising homogenization of milk thistle seeds, NaHCO_3_ heat treatment, and microbial fermentation. The silybin was characterized by high performance liquid chromatography, and the protein was quantified and electrophorized. It was found that the homogenization step was essential for the preparation of protein, and the NaHCO_3_ heat treatment was the crucial step in obtaining silybin. The optimal NaHCO_3_ treatment settings were 1% NaHCO_3_, 60°C, and 3 h, and the optimal strains for microbial fermentation were L131 (*Rummeliibacillus stabekisii*) and RS72 (*Lactobacillus plantarum*). The silybin yield in the fermented milk reached 11.24–12.14 mg/g seeds, accounting for 72.6–78.4% of the total silybin in the milk thistle seeds, and the protein yield reached 121.8–129.6 mg/g seeds. The fermented milk had a slightly sweet yoghurt-like flavor and could be used as a dietary supplement for silybin and protein.

## 1. Introduction

Milk thistle (*Silybum marianum*) is an annual or biennial herbaceous plant belonging to Asteraceae family with a 2000-year history of use [1]. Silymarin is a mixture of flavonolignans contained in the coats of milk thistle seeds, which has a variety of pharmacological activities [2]. Silymarin can prevent liver cell degeneration, promote liver purification, improve detoxification, and also promote the repair of damaged liver cells [3,4,5]. Silymarin mainly scavenges free radicals through anti-lipid peroxidation, increases the level of glutathione (G-SH) in the liver, and therefore exerts a hepatoprotective effect [6,7]. Chemical analysis reveals that silymarin is composed by silybin, isosilybin, silydianin, and silychristin [8]. Silybin and isosilybin account for 60–70% of silymarin. Silybin can significantly inhibit the expression of IL-2, IL-4, IFN-γ, TNF-α in the liver, reduce the levels of alanine aminotransferase and aspartate aminotransferase, and inhibit apoptosis in hepatocyte [6,7,9]. Therefore, the purified silybin and the crude silymarin compounds are important pharmaceutical materials obtained from milk thistle seeds.

There are many methods published for the extraction and purification of silybin from milk thistle seeds. The pharmaceutical industry mainly uses defatted seeds and organic solvents for the extraction of silybin associated with a thermal, ultrasonic, and/or enzymatic process [10,11,12]. For instance, Sun et al. [10] extracted milk thistle with ethyl acetate and ethanol and found the yield of silybin was 10.9 mg/g. Zhang et al. [11] optimized the ultrasonic method of ethanol reflux extraction, and achieved the yield of silymarin as 16.4 mg/g. Wang et al. [12] performed ethanol reflux extraction and obtained 18.3 mg/g silymarin yield. The organic solvents, such as methanol, ethanol, acetone, ethyl acetate, etc., are suitable for pharmaceutical industry whereas they are not suitable for the food industry due to the toxic effects, and in addition, the protein in the milk thistle seeds was fully lost during the extraction of silybin. The milk thistle seeds were found to contain approximately 12.3–16.3 mg/g silybin [10,11,12,13] and 332–360 mg/g protein [14,15,16]. There is no food derived from milk thistle seeds so far, which leaves the possibility of developing a novel food containing both silybin and *Silybum* protein as a dietary and nutritional supplement.

Recent studies suggest that an alkaline solution could be used for silybin extraction. Ren et al. [13] has used 0.5 mol/L NaOH for the extraction of silybin and obtained 2.32 mg/g silybin yield. Li et al. [17] utilized 2 mol/L NaOH to treat defatted seeds of milk thistle, and achieved approximately 10.47 mg/g of crude silymarin. The utilization of NaOH is suitable for pharmaceutical industry whereas it is not suitable for the food industry. We hypothesized that NaHCO_3_ may be superior to the NaOH for the alkaline extraction of silybin in the food process. The reasons are as follows. Firstly, NaHCO_3_ is known as baking soda, which is a mild alkaline, easy to buy, and is an additive that can be used for food processing [18]. Secondly, NaHCO_3_ can interact with the phenolic hydroxyl group of silybin, which improves the hydrolysis and solubility of silybin [8]. Thirdly, NaHCO_3_ can interact with the fatty acids in the milk thistle seeds through saponification, which facilitates the release of silybin from the hydrophobic components [19,20]. Fourthly, NaHCO_3_ can improve the disruption of plant cell walls, which is conducive to the dissolution and release of silybin and protein. However, the high protein and lipid content in the milk thistle seeds makes NaHCO_3_ hard to be effective at a low concentration, and a primary separation step would be helpful to separate protein and lipid from the seed coats prior to the NaHCO_3_ treatment. Furthermore, the bacterial fermentation may improve the release of silybin through enzymatic hydrolysis in vitro, and also introduce new taste and flavor into the fermented liquid [21]. Thus, a three step procedure, comprising homogenization and centrifugation, NaHCO_3_ treatment, and microbial fermentation, could be feasible for the extraction of silybin from the milk thistle seeds, as well as for the recovery of seed protein content.

The objective of this study is to develop a fermented milk containing silybin and protein from the milk thistle seeds, which promotes the dietary and nutritional supplement of silybin and *Silybum* protein, and further suggests a new strategy for the food processing of medical herbs.

## 2. Materials and Methods

### 2.1. Materials and Reagents

Milk thistle seeds were purchased from a local herb store, and were produced in Xi’an, China. The NaHCO_3_ (baking soda) was purchased from a local store. Silybin (S109809, analytical grade), methanol (chromatographical grade), and n-hexane (analytical grade) were purchased from Aladdin Reagent Co., Ltd. (Shanghai, China) for high performance liquid chromatography (HPLC) analysis. Tryptic soy broth (TSB), protein quantification, and electrophoresis kits were purchased from Sangon Biotech Co., Ltd. (Shanghai, China). The fermentation strains were isolated from fermented foods (e.g., rice, tofu, cabbage) by the laboratory, identified through 16S rRNA sequencing, and preserved in the China Center for Type Culture Collection (Wuhan, China) as follows: L131 *Rummeliibacillus stabekisii* (CCTCC M2021182); RS72 *Lactobacillus plantarum* (CCTCC M2021576); RS102 *Streptococcus thermophilus* (CCTCC M2021183); RS92 *Lactobacillus rhamnosus* (CCTCC M2021577); and NZJ *Bacillus subtilis* (CCTCC M2021184).

### 2.2. Extraction with Organic Solvents

Two previous methods used for silybin extraction with organic solvents were performed. Milk thistle seeds were ground into fine powder (particle diameter < 1 mm) using a Scientz-48 grinder (Xinzhi Biological Technology Co., Ltd., Ningbo, China) at 50 Hz for 4 min. Subsamples of ground seed powder were treated by n-hexane overnight at a ratio of 1:10 (*w*/*v*) to remove lipid in the seeds and obtain the defatted seed powder. Method 1 was conducted with methanol solvent following Ren et al. [13]. Briefly, seed powder or defatted powder were soaked in methanol overnight at a ratio of 1:5 (*w*/*v*) and then ultrasonically extracted for 45 min at 30 °C (40 kHz, 100 W) using a G-020S ultrasonic cleaner (Geneng Cleaning Equipment Co., Ltd., Shenzhen, China). The mixture was centrifuged to withdraw the supernatant and repeated for the ultrasonic extraction until the two sectors of supernatant were combined and quantified for the silybin using HPLC analysis. Method 2 was conducted with ethanol solvent following Jia et al. [22]. Briefly, seed powder or defatted powder were mixed with ethanol in a 1:15 *w*/*v* ratio and then sonicated (40 kHz, 120 W) at 60 °C for 60 min using the G-020S ultrasonic cleaner (Geneng). The mixture was centrifuged to obtain the supernatant and was further quantified for silybin using HPLC assay. The seed powder and defatted seed powder were extracted in duplicate for each method with 4 g seeds as starting materials.

### 2.3. Design of the Three Step Procedure

**Figure 1:**
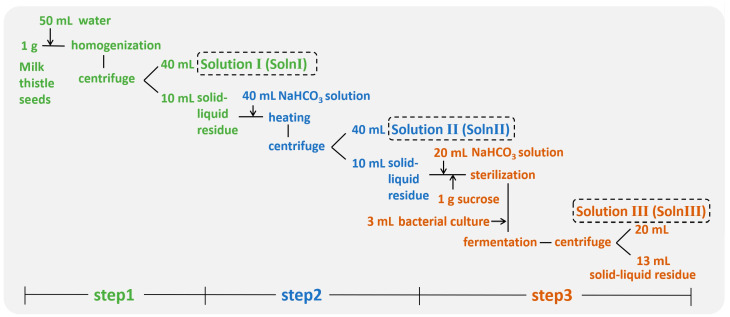
The three step procedure for the preparation of fermented *Silybum* milk. The obtained solutions I, II, and III was combined and sterilized to achieve the final product.

A three step procedure was used to prepare the fermented *Silybum* milk (Figure 1). Firstly, each gram of milk thistle seeds was added with 50 mL water and ground by a colloidal mill (JM50; Jiadeyihai Instruments Ltd., Wenzhou, China) for 5 min. The mixture was centrifuged (4000 rpm, 10 min) to obtain 40 mL supernatant of solution I (Soln I). Secondly, the solid–liquid residue of the first step was added with 40 mL NaHCO_3_ solution, and treated under various conditions of temperature, time, and concentration of NaHCO_3_. The resulting solution was centrifuged (4000 rpm, 10 min) to obtain 40 mL supernatant of solution II (Soln II). Thirdly, the solid–liquid residue of the second step was added with 20 mL NaHCO_3_ solution and 1 g sucrose and further sterilized. The solution was then supplied with 3 mL of bacterial culture and fermented overnight. The resulting solution was centrifuged (4000 rpm, 10 min) to obtain the 20 mL supernatant of solution III (Soln III). Finally, the solutions I, II, and III were combined and sterilized to obtain the fermented *Silybum* milk. Figure 1 shows the volume of solution used per gram seeds, and 4 g milk thistle seeds were used as the starting material in each experiment.

### 2.4. NaHCO_3_ Treatment and Optimization

The NaHCO_3_ treatment was the second step in the three step procedure. The development of NaHCO_3_ treatment was performed as follows: (1) 60 °C, 4% NaHCO_3_ (0.04 g/mL), incubating for 0, 2, 4, 6, and 8 h, respectively; (2) 4 h, 4% NaHCO_3_, incubating at 20, 40, 60, 80, 100 °C, respectively; and (3) 60 °C, 4 h, incubating in 0, 2, 4, 6, 8% NaHCO_3_ solution, respectively. All experiments used water bath with manual shaking for every 15 min. Preliminary results showed that 60 °C was a relatively optimum temperature, whereas the optimum time and concentration of NaHCO_3_ solution were not clear. The optimization of the NaHCO_3_ process was further performed as follows: (4) 60 °C, 2% NaHCO_3_, incubating for 0, 1, 2, 3, 4, 5 h, respectively; and (5) 60 °C, 3 h, incubating in 0, 0.5, 1, 2, 4, 6% NaHCO_3_ solution, respectively. All conditions were performed in duplicate with Soln II quantified for silybin concentration by the HPLC assay. The silybin concentration was transformed to the mass yield with known solution volume and all results of silybin yield were presented in the form of mg/g seeds.

### 2.5. Bacterial Fermentation

The bacterial fermentation was the third step in the three step procedure. To optimize the third step, the second step of the NaHCO_3_ treatment was set at 60 °C, 3 h, 1% NaHCO_3_ (0.01 g/mL) according to the optimal condition identified in Section 2.4. The bacterial culture was obtained with a density > 10^8^ CFU/mL after overnight culturing at 30 °C and 190 rpm rotation speed. The optimization of the third step was carried out as follows: (1) using RS72 strain, fermented in 0, 0.5 and 1% NaHCO_3_ solution for 12 h, 1 d, 2 d, and 3 d, respectively; (2) using 1% NaHCO_3_ solution and 12 h fermentation time, inoculated with the bacterial strain of L131, RS72, RS102, RS103, and NJZ, respectively, associated with the blank control (BK, without bacterial inoculation); and (3) finally, performing a big scale treatment, so that 100 g seeds were processed instead of 4 g engaging with BK, L131, and RS72 condition, respectively. The silybin concentrations in the Solns I, II, and III were quantified using the HPLC, and each condition was conducted in duplicate.

### 2.6. Protein Analysis

The soluble protein contents in the Solns I, II, and III following Section 2.5 (2) were quantified and profiled through gel electrophoresis. The quantification used the Bradford Protein Assay kit (Sangong Bioengineering Co., Ltd., Shanghai, China) following the manufacturer’s instructions. The gel electrophoresis was performed using the denaturing protein electrophoresis method (SDS-PAGE) with 5% concentrated gel and 8% separation gel [23,24]. Then, 4 μL of liquid sample was loaded on the gel and electrophoresed at 180 V for 2 h in a JY-SCZ2+ chamber (Junyidongfang Equipment Co., Ltd., Beijing, China) with PowerPac^TM^ HC electrophoresis instrument (BIO-RAD, Hercules, CA, USA).

### 2.7. HPLC Analysis of Silybin Content

The silybin component in the sample was determined by a SPD-16 HPLC platform (Shimadzu Instrument Manufacturing Co. Ltd., Suzhou, China) equipped with a Zorbax Eclipse XDB-C18 Column (250 mm× 4.6 mm, 5 μm; Agilent Technologies, Santa Clara, CA, USA) following Zhao et al. [25] with modifications. Briefly, dual mobile phases were used in the detection with water as phase A and methanol as phase B. The HPLC was programmed as follows: 0~8 min, phase B increased from 50% to 70%; 8~10 min, phase B increased from 70% to 80%; 10~10.5 min, phase B increased from 80% to 95%; 10.5~12 min, phase B kept at 95%; 12~12.1 min, phase B reduced from 95% to 50%; and 12.1~15 min, phase B kept at 50%. Column temperature was 20 °C; flow rate was 1.2 mL/min; UV detection wavelength was 288 nm. The liquid sample was diluted with methanol, filtered through a 0.45 μm membrane, and examined through the HPLC with 20 μL volume. Silybin standard was prepared as 1 mg/mL in methanol, serially diluted with a 10-fold gradient, and profiled by the HPLC. A standard curve was constructed with the integrated area and standard concentration. The liquid sample was compared with the standard for the HPLC profile and the silybin content was identified through the retention time and quantified using the integrated area and the standard curve.

### 2.8. Statistical Analysis

The silybin yields derived from different conditions were compared by one-way analysis of variance (ANOVA) using multcomp package in the R program (ver3.4.1) [26]. Differences were considered significant at *p* < 0.05.

## 3. Results and Discussion

### 3.1. HPLC Analysis

**Figure 2:**
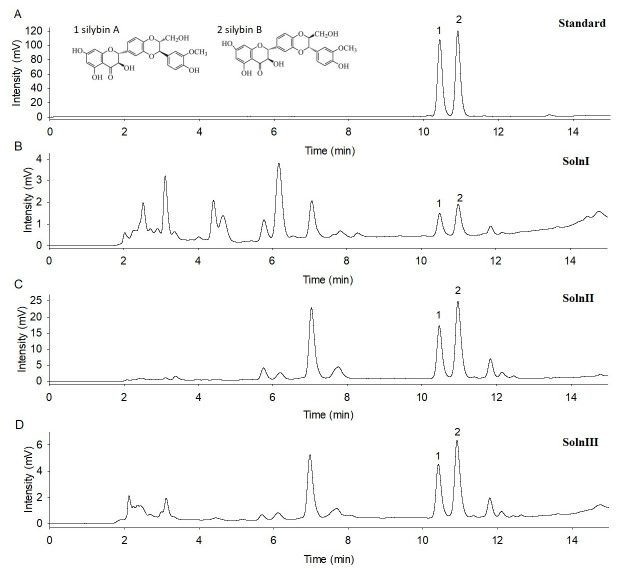
HPLC profiles of silybin standard and solutions I, II, and III samples. (**A**) silybin standard; (**B**) Soln I sample; (**C**) Soln II sample (prepared under conditions of 1% NaHCO_3_, 60 °C, and 3 h); (**D**) Soln III sample (prepared by the inoculation of L131 strain).

There were double peaks in the HPLC profile of the silybin standard, corresponding to silybin A and silybin B (Figure 2A), which is consistent with previous studies [8,27].

The retention times of silybin A and B were 10.431 and 10.912 min, respectively, for the standard (Figure 2A). The HPLC assay revealed that there were silybin peaks in all the Solns I, II and III samples. The retention times were 10.493 and 10.990 min for the Soln I (Figure 2B), 10.469 and 10.966 min for the Soln II (Figure 2C), and 10.420 and 10.918 min for the Soln III (Figure 2D), respectively. Furthermore, the peak intensity indicated that the concentration of silybin content ranked as Soln II > Soln III > Soln I (Figure 2).

### 3.2. Extraction with Organic Solvents

**Figure 3:**
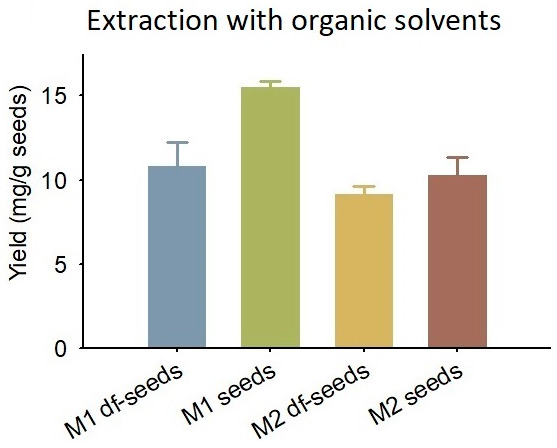
Silybin yields obtained by the extraction method using organic solvents. M1, Method 1; M2, Method 2; df-seeds, defatted seeds. Error bars represent standard deviation.

The silybin yield from Method 1 was 10.84 ± 1.35 mg/g seeds for the defatted seeds and 15.48 ± 0.35 mg/g seeds for the normal seeds (Figure 3). The silybin yield resulted from Method 2 was 9.18 ± 0.43 mg/g seeds for the defatted seeds and 10.3 ± 1.01 mg/g seeds for the normal seeds (Figure 3). Both methods showed that the normal seeds received higher yield than the defatted seeds, which suggested that the oil extraction step by n-hexane may cause silybin loss. Comparing Method 1 and Method 2, Method 1 obtained a higher yield than Method 2, which could be partially explained by the solvent difference, that the methanol used in Method 1 was more efficient than the ethanol used in Method 2 for the silybin extraction purpose [13]. Several studies have reported the yield of silybin or silymarin with an organic extraction method: Zhang et al. [11] applied an ultrasound-assisted ethanol reflux method and achieved an extraction yield of 16.4 mg/g for silymarin; Ruan et al. [28] performed an ultrasound-assisted ethanol and ammonium sulfate biaqueous extraction and obtained a silybin yield of 10.18 mg/g; Sun et al. [10] utilized ethyl acetate for the extraction and used ethanol for the recrystallization, and obtained a silybin yield of 10.9 mg/g; Wang et al. [12] performed a silybin extraction with ultrasound-assisted ethanol method and reported the yield as 16.02 mg/g. The silybin yield of 15.48 ± 0.35 mg/g seeds (Method 1, normal seeds) in this study was aligned with previous reports. Therefore, the current milk thistle seeds were considered embracing 15.48 ± 0.35 mg/g silybin content.

### 3.3. NaHCO_3_ Thermal Treatment

**Figure 4:**
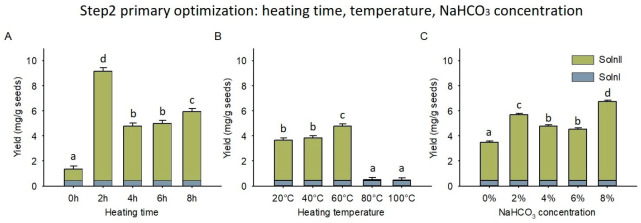
Silybin yields under various NaHCO_3_ thermal conditions (primary optimization). (**A**) conditions of 60 °C, 4% NaHCO_3_, and varied heating times; (**B**) conditions of 4 h, 4% NaHCO_3_, and varied heating temperatures; (**C**) conditions of 60 °C, 4 h, and varied NaHCO_3_ concentrations. Error bars represent standard deviation. Different letters indicate significant differences. Soln I, Solution I; Soln II, Solution II.

The silybin yield in Soln I was 0.434 mg/g seeds as detected by the HPLC. The silybin yield in Soln II exhibited significant difference among varied NaHCO_3_ thermal conditions (Figure 4). The heating time comparison revealed that 2 h was superior to the other times under 60 °C and 4% NaHCO_3_ (*p* < 0.001), that the silybin yield reached 8.75 mg/g seeds (Figure 4A). The heating temperature comparison indicated that 60 °C outperformed the other temperatures under 4 h and 4% NaHCO_3_ (*p* < 0.001), and the silybin yield achieved 4.35 mg/g seeds (Figure 4B). The NaHCO_3_ concentration assay suggested that 8% NaHCO_3_ was better than the other concentrations under 60°C and 4 h (*p* < 0.001), that the silybin yield gained 6.33 mg/g seeds (Figure 4C). Therefore, the heating time of 2 h, heating temperature of 60 °C, and NaHCO_3_ concentration of 8% were the optimal conditions in the preliminary determination of NaHCO_3_ thermal conditions. However, the advantage of 60 °C was clear in the thermal condition comparison, whereas the heating time and NaHCO_3_ concentration could be further optimized.

**Figure 5:**
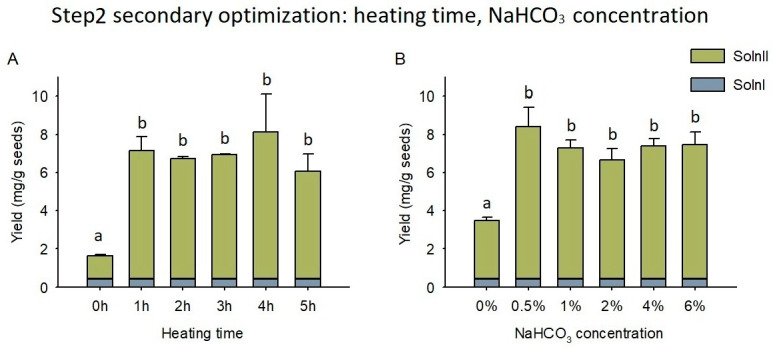
Silybin yields under various NaHCO_3_ thermal condition (secondary optimization). (**A**) conditions of 60 °C, 2% NaHCO_3_, and varied heating times; (**B**) conditions of 60 °C, 3 h, and varied NaHCO_3_ concentrations. Error bars represent standard deviation. Different letters indicate significant differences. Soln I, Solution I; Soln II, Solution II.

The second round of NaHCO_3_ optimization focused on the possibility of using long-time treatment under 60 °C with low concentrations of NaHCO_3_. The time groups of 1–5 h showed similar silybin yield of 5.65–7.69 mg/g seeds under 60 °C and 2% NaHCO_3_ condition, which were all significantly higher than the 0 h group (1.19 mg/g seeds, Figure 5A). The 1 h and 3 h group obtained silybin yield of 6.73 ± 0.73 and 6.53 ± 0.04 mg/g seeds, respectively. The higher reproducibility of the 3 h than the 1 h group made the 3 h the optimum condition. Furthermore, the concentration groups of 0.5%–6% NaHCO_3_ exhibited a similar silybin yield of 6.23–7.98 mg/g seeds under 60 °C and 3 h heating, which were all significantly higher than the 0% NaHCO_3_ group of 3.04 mg/g seeds (Figure 5B). The 0.5% and 1% NaHCO_3_ group reached a silybin yield of 7.98 ± 1.04 and 6.88 ± 0.40 mg/g seeds, respectively. Additionally, the higher reproducibility of 1% than 0.5% NaHCO_3_ condition made the 1% NaHCO_3_ the optimal condition. Therefore, the optimal condition for NaHCO_3_ thermal treatment was established as 60 °C, 3 h, and 1% NaHCO_3_ with the silybin yield of 6.88 ± 0.40 mg/g seeds in the Soln II.

The current study used a step-by-step optimization procedure for the silybin yield improvement, i.e., from temperature condition to the duration time and NaHCO_3_ concentrations, respectively, instead of using the response surface methodology (RSM) [29]. The reasons were as follows: (1) the temperature was found to be the critical parameter for the silybin extraction with NaHCO_3_ treatment, since high temperature, e.g., 80 °C and 100 °C, caused great degradation of silybin in the Soln II (Figure 4B). Although temperatures over the 60–80 °C range may lead to a higher yield than 60 °C, the risk of thermal decomposition of silybin was also raised [30]. Therefore, the current strategy used the mild thermal condition of 60 °C instead of further optimizing the temperature to reduce the risk of silybin thermal degradation. (2) The thermal duration times and NaHCO_3_ concentrations resulted in an optimal range instead of certain points for a comparable silybin yield (Figure 5). Therefore, the RSM strategy may lead to a bias that not only one condition could result in a superior yield. Instead, the reproducibility of the extraction procedure was important, that the 3 h duration time and 1% NaHCO_3_ were selected due to the stable silybin extraction performance. (3) The simplicity and feasibility of the technical parameters were of significance since the current method aimed to be applied in the food industry. The parameters of 60 °C, 3 h, and 1% NaHCO_3_ were easy and feasible in a food factory, which fulfilled the application needs. (4) Finally, the silybin that remained in the Soln II residue could be further extracted in Soln III.

A few studies have also used alkaline solution for the silybin extraction from the defatted milk thistle seeds. Ren et al. [13] reported that the treatment with 0.5 mol/L NaOH resulted in a silybin yield of 2.32 mg/g. Li et al. [17] found that 2 mol/L NaOH extraction associated with ultrasonic treatment resulted in a silymarin yield of 10.47 mg/g. The current study used 1% NaHCO_3_, i.e., 0.119 mol/L NaHCO_3_, which was milder than the previous 0.5 mol/L or 2 mol/L NaOH solution, whereas it achieved a comparable yield of silybin (6.88 ± 0.40 mg/g seeds). This high extraction efficiency could be derived from four reasons. Firstly, the current study utilized a three step extraction procedure, for which a large amount of seed protein was collected and removed in the first homogenization step, which allowed the NaHCO_3_ to mainly interact with the ground seed coats instead of the seed protein in the second step. Secondly, the NaHCO_3_ interacted with the phenolic hydroxyl group of silybin, which enhanced the solubility of silybin. Thirdly, the current procedure utilized a heating method incorporated with NaHCO_3_ treatment, which facilitated the saponification of seed fatty acids. Fourthly, the NaHCO_3_ improved the disruption of plant cell walls, which facilitated the release of silybin.

### 3.4. Microbial Fermentation

**Figure 6:**
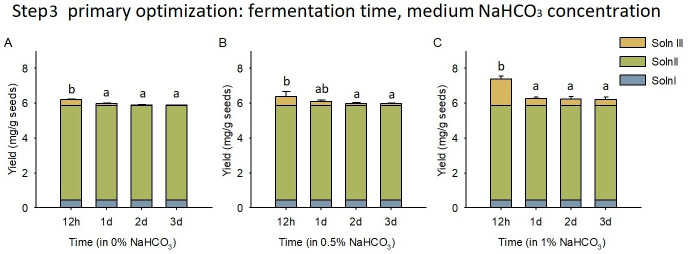
Silybin yields obtained in the third step with varied NaHCO_3_ concentration and fermentation time. (**A**), fermentation within 0% NaHCO_3_; (**B**), fermentation within 0.5% NaHCO_3_; (**C**), fermentation within 1% NaHCO_3_. The fermentation used RS72 *Lactobacillus plantarum* strain. Error bars represent standard deviation. Different letters indicate significant differences. Soln I, solution I; Soln II, solution II; Soln III, solution III.

The NaHCO_3_ concentration in the broth and the fermentation time had significant effects on the silybin yield in the Soln III. The 1% NaHCO_3_ broth resulted in a higher silybin yield than the 0% and 0.5% NaHCO_3_ broth (*p* < 0.001, Figure 6). The 12 h fermentation time achieved higher silybin yield than the 1 d, 2 d, and 3 d time (*p* < 0.001, Figure 6), which suggested that bacteria may utilize silybin during the long-time fermentation. Engaging with the RS72 *Lactobacillus plantarum* strain, the fermentation results indicated that the 1% NaHCO_3_ broth and 12 h fermentation time were the optimal conditions for the third step, and the silybin yield achieved 1.50 ± 0.19 mg/g seeds in Soln III under this condition (Figure 6C).

**Figure 7:**
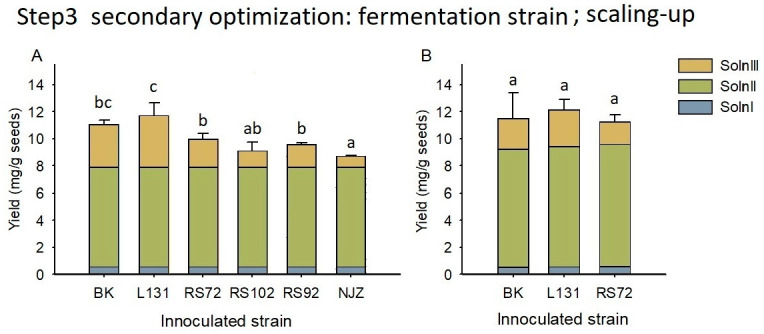
Silybin yields in the three step processing of milk thistle seeds with varied fermentation bacteria. (**A**) blank control (BK) and five strains (L131, RS72, RS102, RS92, NJZ) were used in the fermentation step. (**B**) blank control (BK) and two strains (L131, RS72) were used in the scaling-up processing of milk thistle seeds (100 g). Error bars represent standard deviation. Different letters indicate significant differences. Soln I, solution I; Soln II, solution II; Soln III, solution III.

Using the fermentation condition of 1% NaHCO_3_ broth and 12 h time, varied bacterial strain resulted in different silybin yields in the fermentation step (Figure 7). The silybin yields in Soln III were found as follows: BK (3.16 ± 0.34 mg/g seeds), L131 (3.83 ± 0.96 mg/g seeds), RS72 (2.07 ± 0.42 mg/g seeds), RS102 (1.22 ± 0.67 mg/g seeds), RS92 (1.69± 0.14 mg/g seeds), and NJZ (0.82 ± 0.07 mg/g seeds). The silybin yields in the Soln I and Soln II were 0.549 and 7.34 mg/g seeds, respectively. Therefore, the integral silybin yields following the three step procedure were achieved as: BK (11.05 mg/g seeds), L131 (11.71 mg/g seeds), RS72 (9.96 mg/g seeds), RS102 (9.10 mg/g seeds), RS92 (9.57 mg/g seeds), and NJZ (8.70 mg/g seeds) (Figure 7A). Except for the BK, the Soln III that resulted from the bacterial fermentation had a slightly sweet taste and yoghurt-like flavor, while the Soln III of BK exhibited a slightly bitter taste without a special flavor. The BK, L131, and RS 72 were used for scaling-up processing (100 g) due to the relative higher silybin yields than other strains. The silybin yields for Soln I, II, and III following the scaling-up processing were found as 0.528, 8.69, and 2.27 mg/g seeds for the BK (i.e., 4.5%, 75.6%, 19.8% of the total yield), 0.548, 8.89, and 2.70 mg/g seeds for the L131 (i.e., 4.5%, 73.2%, 22.3% of the total yield), and 0.570, 9.03, and 1.65 mg/g seeds for the RS72 (5.1%, 80.3%, 14.6% of the total yield), respectively. The total yields of silybin resulted from the scaling-up processing were 11.49 ± 1.93 mg/g seeds for the BK, 12.14 ± 0.78 mg/g seeds for the L131, and 11.24 ± 0.54 mg/g seeds for the RS72, respectively (Figure 7B).

The five bacteria (L131, RS72, RS102, RS92, NJZ) used in this study are all food fermentation strains, which are commonly used in the food industry for dairy, rice, and vegetable fermentation [21]. The silybin yield in Soln III suggested the L131 and RS72 were optimal bacterial strains for the fermentation step. The RS102, RS92, and NJZ strain may consume silybin during the fermentation (Figure 7A). Although the silybin yield of L131 and RS72 did not improve significantly compared with BK, the liquid flavor did upgrade a lot, being slightly sweet and with a yoghurt-like taste. For both the small scale (4 g) and big scale (100 g) processing, the silybin yield in the Soln I, II, and III ranked as Soln II > Soln III > Soln I (Figure 7), and the second NaHCO_3_ thermal step achieved the highest silybin yield, accounting for 73–80% of the total yield, while the bacterial fermentation step accounted for 15–22% of the total yield, and the homogenization step represented 4–5% of the total yield. The scaling-up processing did not lower the silybin yield, whereas elevating the processing efficiency probably due to the reduction of operational or material loss, which suggests the current three step method could be scaled up. The final silybin yield derived from L131 or RS72 fermentation achieved 12.14 ± 0.78 mg/g seeds and 11.24 ± 0.54 mg/g seeds, respectively (Figure 7B, 100 g scale). These yields were comparable to the yield of 10.9–16.4 mg/g in the previous reports engaging with chemical extraction methods [10,11,12,13]. The current milk thistle seeds were found to contain 15.48 mg/g silybin using the methanol extraction method (Section 3.2). The total silybin yields of the L131 and RS72 procedure accounted for 78.4% and 72.6% silybin contained in the milk thistle seeds, respectively, which suggests the current three step processing strategy achieved good efficiency for the silybin extraction through food processing technology.

### 3.5. Protein Analysis

**Figure 8:**
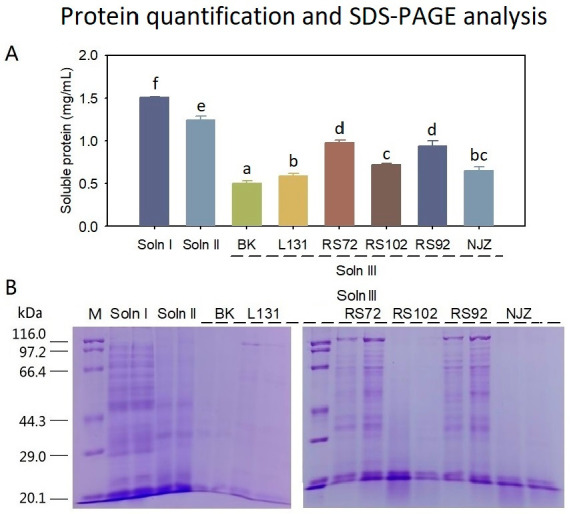
Protein quantification analysis (**A**) and denaturing gel electrophoresis assay (**B**). (**A**) different letters indicate significant differences. Soln I, Solution I; Soln II, Solution II; Soln III, solution III; M, protein molecular weight marker; BK, blank control; and L131, RS72, RS102, RS92, NJZ, fermentation strains.

Soluble protein was detected in all the Soln I, II, and III samples, with the concentration ranked as Soln I > Soln II > Soln III (Figure 8A). The Soln I contained 1.51 mg/mL soluble protein, the Soln II obtained 1.24 mg/mL of soluble protein, and the Soln III had 0.50–0.98 mg/mL soluble protein (Figure 8A). The bacterial fermentation resulted in more protein released in the Soln III than the BK condition (*p* < 0.05), and the L131 (0.59 mg/mL), RS72 (0.98 mg/mL), RS102 (0.72 mg/mL), RS92 (0.94 mg/mL), NJZ (0.65 mg/mL) all showed a higher protein concentration than the BK (0.50 mg/mL). The PAGE gels aligned with the protein quantification results, showing that the Soln I sample contained more protein bands than Soln II and Soln III with the protein molecular weight ranging from 0–116 kDa (Figure 8B). The Soln III samples of RS72, RS92, and L131 showed more protein bands than the BK with wide distribution of protein size. Considering every gram of milk thistle seeds resulted in 40 mL Soln I, 40 mL Soln II, and 20 mL Soln III, the soluble protein yields derived from three step processing were found as 60.4 mg/g (Soln I), 49.6 mg/g (Soln II), and 11.8 mg/g (L131) or 19.6 mg/g (RS72) (Soln III), respectively. In total, the three step processing method retrieved 121.8 and 129.6 mg/g soluble protein for the L131 and RS72 fermentation, respectively. There was approximately 332–360 mg/g total protein contained in the milk thistle seeds [14,15,16], among which 58% protein was soluble. Therefore, the current three step procedure achieved 33.8–39.0% of the total protein in seeds, accounting for 58.3–67.3% of the total soluble protein, which highlights that the current method is efficient in the extraction of seed protein.

### 3.6. Summary of the Three Step Procedure

**Figure 9:**
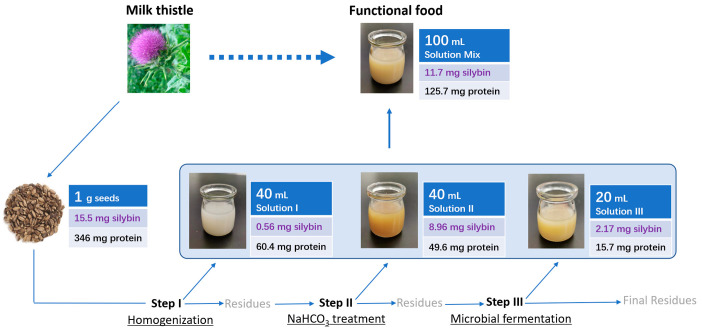
Summary of the three step procedure for the preparing of the *Silybum*-fermented milk. The milk contained the silybin and protein from the milk thistle seeds. The silybin and protein yields were derived from the average data gained through the scaling-up processing of the L131 and RS72 fermentation.

The current study developed a three step procedure for the preparation of a fermented milk containing silybin and protein from the milk thistle seeds. The procedure included the homogenization of milk thistle seeds, NaHCO_3_ heat treatment, and microbial fermentation (Figure 9). The optimal conditions for the NaHCO_3_ treatment were 1% NaHCO_3_, 60 °C, and 3 h time, and the optimal fermentation strains for the microbial fermentation were L131 (*Rummeliibacillus stabekisii*) and RS72 (*Lactobacillus plantarum*). The silybin yield in the fermented milk achieved 11.24–12.14 mg/g seeds, accounting for 72.6–78.4% of the total silybin in seeds, and the soluble protein yield reached 121.8–129.6 mg/g seeds, accounting for 33.8–39.0% of the total protein in seeds. The fermented milk had slightly sweet taste and a yoghurt-like flavor, which could be used for a dietary supplement of silybin and protein.

## 4. Conclusions

The current study achieved a fermented *Silybum* milk containing silybin and protein. The preparation method included three steps, namely homogenization of milk thistle seeds, NaHCO_3_ heat treatment, and microbial fermentation treatment. The homogenization step was important for the preparation of the protein, and the NaHCO_3_ heat treatment was the key procedure in obtaining silybin. Under optimal conditions, the silybin yield in the fermented milk reached 11.24–12.14 mg/g seeds, accounting for 72.6–78.4% of the total silybin in seeds, while the soluble protein yield achieved 121.8–129.6 mg/g seeds, accounting for 33.8–39.0% of the total protein in seeds. The current study provides a novel strategy for the food processing of medical herbs. Further studies can be carried out for the human health effect of the fermented milk and also for the preservation technology of the fermented milk in long-term storage.

## Data Availability

Data are available upon request to the corresponding author.
